# Modulation of Lipid Profile and Lipoprotein Subfractions in Overweight/Obese Women at Risk of Cardiovascular Diseases through the Consumption of Apple/Berry Juice

**DOI:** 10.3390/antiox11112239

**Published:** 2022-11-14

**Authors:** Marta Habanova, Maria Holovicova, Hana Scepankova, Marta Lorkova, Jan Gazo, Martina Gazarova, Carlos A. Pinto, Jorge A. Saraiva, Leticia M. Estevinho

**Affiliations:** 1 The Institute of Nutrition and Genomics, Slovak University of Agriculture in Nitra, Trieda Andreja Hlinku 2, 94976 Nitra, Slovakia; 2 The AgroBioTech Research Center, Slovak University of Agriculture in Nitra, Trieda Andreja Hlinku 2, 94976 Nitra, Slovakia; 3 LAQV-REQUIMTE, Chemistry Department, Campus Universitário de Santiago, University of Aveiro, 3810-193 Aveiro, Portugal; 4 Centro de Investigação de Montanha, Instituto Politécnico de Bragança, 5300-252 Bragança, Portugal; 5 The Institute of Plant and Environmental Sciences, Slovak University of Agriculture in Nitra, Trieda Andreja Hlinku 2, 94976 Nitra, Slovakia; 6 Laboratório para a Sustentabilidade e Tecnologia em Regiões de Montanha, Campus de Santa Apolónia, Instituto Politécnico de Bragança, 5300-253 Bragança, Portugal

**Keywords:** overweight, obesity, antioxidants, berries, juice, low-density lipoprotein, cholesterol, atherogenic subfraction, intervention

## Abstract

Polyphenol-rich foods protect the cellular systems of the human body from oxidative damage, thereby reducing the risk of chronic diseases such as cardiovascular disease (CVD). We investigated the effect of phenolic-rich apple/berry juice (chokeberry, blueberry, and cranberry) on lipidemic profiles in overweight/obese women. The 6 week single-arm pre–post intervention study involved 20 women (mean age 52.95 ± 5.8 years, body mass index ≥25 kg/m^2^, and ≥1 CVD risk factors) consuming 300 mL/day of the apple/berry juice. Lipid profile, low-density lipoprotein (LDL) subfractions assessed using Lipoprint^®^ electrophoresis, and other parameters related to cardiovascular risk (C-reactive protein, glucose, blood pressure) were analyzed before and again after the intervention in the monitored group of women. High-density lipoprotein cholesterol (HDL-C) increased from 1.30 ± 0.29 to 1.55 ± 0.32, magnesium from 0.85 ± 0.03 to 0.90 ± 0.05, and total antioxidant status from 1.68 ± 0.08 to 1.81 ± 0.10. The LDL/HDL ratio significantly decreased from 3.40 ± 0.99 to 2.66 ± 0.63 mmol/L, and the glucose from 5.50 ± 0.72 to 5.24 ± 0.74 mmol/L. However, the hs-CRP did not change significantly. Women with atherogenic subfractions LDL3-7 at baseline (*n* = 6) showed a significant reduction from 0.45 ± 0.19 to 0.09 ± 0.07 mmol/L. Overweight/obese women may benefit from apple/berry juice as part of a healthy lifestyle to improve their lipid profile, and thus, contribute to cardiovascular health.

## 1. Introduction

Overweight and obesity have consistently been associated with an increased risk of CVD, particularly coronary artery disease (CAD) and heart failure [1]. Overweight and obesity are defined as an abnormal or excessive fat accumulation in the body that may impair health [2]. Obese subjects are primarily at an increased cardiovascular risk due to visceral adiposity because the pathophysiological processes that lead to body fat accumulation eventually lead to fat accumulation in other body compartments than subcutaneous tissue. Moreover, visceral adiposity is accompanied by an increased inflammatory response that contributes to all stages of the formation of vascular lesions and subsequent CVD, and is maintained and impaired by several risk factors such as an unhealthy diet and lifestyle, smoking, hyperlipidemia/hypercholesterolemia, hypertension, autoimmune diseases, etc. [3,4,5]. 

The global increase in the prevalence of overweight and obesity, and the elevated risk of CVDs has become a significant public health concern among women [6]. According to a recent study, the risk of CVD is 40 percent higher for overweight women, 60 percent higher for women with general obesity, and 30 percent higher for women with abdominal obesity compared to men [7]. The mechanisms via which obesity leads to cardiovascular risk in women are discrepant between their premenopausal, pregnancy, and postmenopausal phases of life [8]. Women after middle age, particularly postmenopausal women, tend to have a deteriorated lipid profile that becomes more atherogenic than their premenopausal counterpart. After menopause, total cholesterol (T-C) and low-density lipoprotein cholesterol (LDL-C) usually increase, and these changes are accompanied by a decrease in HDL-C and an increase in triglycerides (TG). In addition to these major lipid abnormalities, modifications in the size and density of these lipoprotein particles are expected to happen due to the declining levels of estrogen in postmenopausal women [9,10,11]. Additionally, aging is associated with chronic low-grade inflammation and visceral adiposity, which alters lipid profiles in a proatherogenic manner [4]. 

Atherogenic dyslipidemia is characterized by an abnormal amount of blood lipids, particularly elevated levels of LDL-C, TG, sdLDL (LDL3-7 subfractions), and decreased serum levels of HDL-C [12]. The sdLDL have been recognized as an emerging risk factor for CVD. If the clinical decision is made to detect and measure the sdLDL subfractions, their presence is a better indicator of atherogenic dyslipidemia and metabolic syndrome [12] because it is more atherogenic than LDL [13]. Indeed, the sdLDL particles are small and have greater arterial entry and retention, higher susceptibility to oxidation, as well as a reduced affinity for the LDL receptor [14]. Nevertheless, a major lipid alteration in patients with CAD, peripheral arterial disease, abdominal aortic aneurysm, diabetes, metabolic syndrome, and other categories of patients with high cardiovascular risk have been observed [15]. Monitoring and controlling the lipid profile are considered necessary to reduce the risk of CVD [16]. Moreover, there is recent evidence that treatment of inflammation, either prevention or reversal, is the key therapeutic aim in CVD [17]. 

A dietary strategy based on heart-healthy eating patterns is recommended to improve CVD risk factors, such as dyslipidemia [18]. Epidemiological studies have demonstrated that polyphenol-rich fruit has a positive effect in reducing the risk of CVD [19] through antioxidant, anti-inflammatory, and even selective antimicrobial properties [20]. Several studies demonstrated that the consumption of berry fruits, berry-based functional foods, and dietary supplements can reduce inflammatory angiogenesis and achieve optimal plasma lipoprotein profiles, thus, reducing CVD risk [21,22,23,24,25,26,27,28]. Various types of berries, including strawberry, raspberry, blackberry, blueberry, cranberry, and chokeberry, are rich dietary sources of bioactive compounds. Due to their high antioxidant characteristics and high level of phenolic compounds, they are of significant interest to nutritionists and food engineers as functional food additives [29]. 

However, no clinical studies have been carried out regarding the relationship between the intake of pure berry-enriched fruit juice and the modulation of the lipid profile and atherogenic LDL subfractions in overweight/obese women with associated CVD risk factors. 

The present paper aims to study the hypothesis that the regular consumption of apple/berry juice (chokeberries, blueberries, and cranberries) as a rich source of antioxidant phenolics may have beneficial effects on modulating CVD risk factors (e.g., increasing HDL-C, decreasing T-C, LDL-C, TG, and the atherogenic subfraction LDL3-7 in middle-aged women at risk of CVD). To test this hypothesis, women from the ranks staff of the Slovak University of Agriculture volunteered to regularly ingest the apple/berry juice for 6 weeks, and their lipid profile (T-C; LDL-C, HDL-C, TG), LDL subfractions, and other biochemical parameters such as high-sensitivity C-reactive protein (hs-CRP), glucose, and total antioxidant status (TAS) were monitored before and after the intervention. 

## 2. Materials and Methods

### 2.1. Participants and Study Design

The present study carried out a single-arm pre–post intervention study [30] that measured the effect of the regular consumption of apple/berry juice on changes in anthropometric parameters and the modulation of lipid profiles in overweight/obese women with associated CVD risk factors. A total of 53 female volunteers from the staff of the Slovak University of Agriculture were screened for eligibility of their participation based on the inclusion and exclusion criteria. The volunteers had to meet the following inclusion criteria: willingness to participate in a 6-week interventional program; overweight/obese based on anthropometric parameters: BMI (≥25 kg/m^2^); one or more than one CVD risk factor, such as an abnormal lipid profile (TC ≥ 5.20 mmol/L, LDL-C ≥ 3.4 mmol/L, HDL-C ≤ 1.03 mmol/L, and/or TG ≥ 1.70 mmol/L), hypertension (systolic blood pressure ≥140 mm Hg, or diastolic blood pressure ≥90 mm Hg), and/or presence of atherogenic small-dense LDL (LDL3-7 subfractions); aged 40–60 years; stable body weight (±3 kg) during the last 3 months; and alcohol consumption of ≤30 g/day. The exclusion criteria included an inability to give informed consent; chronic diseases (i.e., CVD, inflammatory diseases, diabetes, cancer, and allergy); thyroid abnormalities; active liver disease; use of corticosteroids; self-reported high concentration of fat or lipid in the blood and high blood pressure treated with drugs (known from previous medical examinations); use of cholesterol-lowering medications or supplements; pregnancy; tobacco, alcohol, or drug addiction; use of antacids or laxatives at least once a week; an irregular or unbalanced dietary pattern; food intolerance or allergy to the polyphenols; intake of any berries, berry products, and nutritional supplements (vitamins, minerals, antioxidants, and flavonoids); and parallel participation in other dietary intervention studies. 

This study was conducted according to the guidelines of the Declaration of Helsinki and approved by the Slovak University of Agriculture in Nitra, Department of Human Nutrition, Slovakia; and by the Ethical Committee of the Specialized Hospital of St. Svorad Zobor in Nitra, Slovakia (Study No. 01/0906/2015). All participants had written informed consent before the enrolment, and before the intervention itself, they were informed about the possible risks.

### 2.2. Intervention

**Figure 1:**
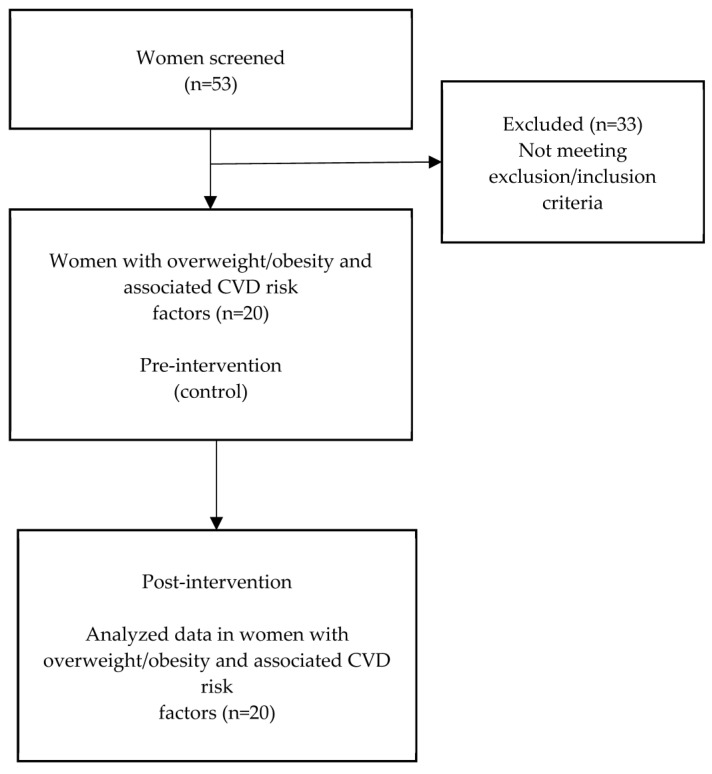
Flowchart of the pre–post intervention and completion of participant selection.

A group of 20 adult women (mean age 52.95 ± 5.8 years) participated in a 6-week pre–post interventional program (Figure 1). The women involved in the study were instructed to consume 300 mL/day of the apple/berry mixed juice for 6 weeks as part of their regular diets. During the study, the women were asked to follow their habitual diet and do their usual physical activity, and not change their usual eating habits or lifestyle. Before (pre) and after (post) the 6 weeks of the study, the blood serum of each participant was analyzed relative to its lipid profile: TC, LDL-C, HDL-C, TG; lipoprotein subfraction profile such as the very-low-density lipoprotein (VLDL) fractions, the intermediate-density lipoprotein (IDL), and the LDL with subfractions 1 and 2 as large LDL (LDL1, LDL2) and atherogenic subfractions LDL3-7 as small-dense LDL (sdLDL); and other biochemical parameters such as TAS, magnesium (Mg), and hs-CRP. We compared these parameters pre-and post-intervention to demonstrate a possible causal relationship, according to the methodology described by Harris et al. (2006) [31]. 

The juice consisted of chokeberry (*Aronia melanocarpa* Michx. Elliott), blueberry (*Vaccinium corymbosum* L.), cranberry (*Vaccinium vitis-idaea* L.), and apple (*Malus* ssp.) in the proportion of 25:15:10:50, respectively. To ensure microbial safety, the apple/berry juice was heat pasteurized at 72 °C for 30 min. No water, sugar, and food preservatives were added, and the bottles of juice were kept under refrigeration. The concentrations of total phenolics, total anthocyanins, and individual phenolics of the apple/berry juice mixture, as well as the antioxidant activity, were quantified.

The total phenolic content was determined according to the Folin–Ciocalteu method [32]; the total anthocyanins content was evaluated as described by [33]. Individual phenolic compounds, such as rutin, quercetin, kaempferol, chlorogenic acid, rosmarinic acid, caffeic acid, and gallic acid were quantified using the analytical method HPLC (HPLC Waters Breeze, Waters Corporation, Milford, MA, USA) and adapted according to the methodology used by [34]. The antioxidant activity was determined based on the stable synthetic free radical 2,2-diphenyl-1-picrylhydrazyl (DPPH) [35]. The method was slightly modified as reported by [36], and the results were expressed as the percentage of inhibition of the DPPH radical. 

### 2.3. Anthropometric Measurements

Anthropometric measurements were performed at the beginning of the research (week 0; pre) and the end (6 weeks; post) by trained personnel. Body height (cm) was measured in the upright standing position, without shoes, by outpatient electronic medical scales (Tanita WB-3000, Tanita Co., Tokyo, Japan). Bodyweight and body composition, such as percent of body fat (PBF) and skeletal muscle mass (SMM), were diagnosed by multi-frequency bioelectrical impedance analysis (MFBIA) using the InBody 720 (Biospace Co., Seoul, Korea). The BMI was calculated by dividing the body weight in kilograms by the square of the height in meters. Women with a BMI of 25–30 kg/m^2^ were categorized as overweight and those with a BMI ≥ 30 kg/m^2^ were categorized as obese. The waist-to-hip ratio (WHR) was calculated as the waist measurement divided by the hip measurement [37]. Systolic and diastolic blood pressure (in mm Hg) and pulse were measured twice using a Sphygmomanometer DM-3000 (Nihon Seimitsu Sokki Co., Nakago Shibukawa Gunma, Japan) in a remained seated and relaxed position (participants rested for at least 15 min before each measurement).

### 2.4. Preparation of Blood Samples

The collections of blood samples were taken pre-and post-intervention. The venous blood from the peripheral vein of the elbow socket was collected in the morning after 8 h of fasting in a standard manner using 2.5 mL of ethylenediaminetetraacetic acid (EDTA) solution and in a 7.5 mL serum gel tube. Once blood serum had been separated, the samples were stored at 80 °C until clinical parameters were determined. 

### 2.5. Clinical Parameters

The fasting lipid profile in blood serum (TC, LDL-C, HDL-C, TG), hs-CRP, glucose, TAS, and Mg were determined from thawed serum samples using a biochemical analyzer Biolis 24i Premium (Tokyo Boeki Machinery, Tokyo, Japan) and commercially available kits DiaSys (Diagnostic System GmbH, Holzheim, Germany) and Randox (Randox Laboratories Ltd., Crumlin, UK). Biochemical parameters were determined using the application protocol provided by the supplier (Ecomed, Zilina, Slovakia) with modifications made when necessary. The lipid profile obtained for each participant was then compared to the reference values of “The National Cholesterol Education Program: Adult Treatment Panel III (NCEP ATP III)” [12]. The conversion of units from the mg/dL to mmol/L was carried out using the following conversion factors: (1) mg/dL × 0.02586 = mmol/L for the total cholesterol, HDL cholesterol, and LDL cholesterol; and (2) mg/dL × 0.01129 = mmol/L for triglycerides [38].

The lipoprotein subfractions VLDL, IDL 1–3, LDL1, LDL2, and LDL 3–7 were determined in blood serum using the analyzer Lipoprint^®^ (Quantimetrix Corp., Redondo Beach, CA, USA) with Quantimetrix Lipoprint System LDL Subfractions Kit “Lipoprint LDL Kit” (Quantimetrix, Redondo Beach, CA, USA), according to the procedure provided by the manufacturer. This method uses linear electrophoresis on a nondenaturing polyacrylamide gel to separate and quantify lipoprotein subfractions. Based on the size of the LDL subtraction particles, the Lipoprint^®^ reports the LDL phenotype as non-atherogenic phenotype A (≥26.8 nm), intermediate phenotype AB (26.53–26.79 nm), and atherogenic phenotype B (≤26.5 nm). 

### 2.6. Statistical Analysis

Data were expressed as the mean ± standard deviation (SD) and as median and interquartile ranges. Statistical analyses were carried out using Microsoft^®^ Excel^®^ (Microsoft Corporation, Redmond, WA, USA) and the data were analyzed by *t*-test. Differences were considered statistically significant at *p* < 0.05. 

## 3. Results

### 3.1. Characteristics of Study Participants

A group of 20 women ranging in age from 41 to 60 years with a mean age of 52.95 ± 5.8 years participated in the present study. Table 1 shows the baseline characteristics of the participants in the study. Based on the BMI index, the study population was comprised of 3 participants with obesity (BMI ≥ 30 kg/m^2^) and 17 overweight participants (BMI 25–29.9 kg/m^2^). The values of PBF and WHR were beyond the standard range in all women. All subjects had insufficient SMM. 

Regarding the metabolic characteristics, all participants had high/higher borderline T-C (6.29 ± 0.99) and LDL-C (4.19 ± 0.83). Based on the LDL/HDL ratio, 13 women were at the risk level (above 3.0 mmol/L) for CVD. Furthermore, atherogenic sdLDL subfractions (LDL3-7) were found in six women. The inflammatory biomarker hs-CRP was detected in four women at a level associated with a high risk of CVD (above 3 mg/L). Baseline data showed that all women enrolled in the study were overweight/obese with one or more risk-associated CVD factors.

### 3.2. Phenolic Compounds in Apple/Berry Juice

The amount of the total phenolics, total anthocyanins, and individual phenolics (i.e., rutin, quercetin, kaempferol, chlorogenic acid, rosmarinic acid, caffeic acid, and gallic acid) in the apple/berry juice are shown in Table 2. The total phenolics in the apple/berry juice were 286.25 ± 8.41 µg/mL, and rutin (64.64 ± 7.12 µg/mL), quercetin (20.77 ± 0.80 µg/mL), and chlorogenic acid (6.80 ± 0.62 µg/mL) were the phenolics with the highest concentrations. These compounds engaged in effective free radical scavenging [36] and contributed to the high antioxidant activity (73.24 ± 1.85%) quantified. 

### 3.3. Changes in Anthropometric Parameters, Blood Pressure, and Lipid Profile

The data of the anthropometric parameters, blood pressure, blood serum lipid profile (T-C, HDL-C, LDL-C, and TG), and other biochemical parameters (TAS, Mg, glucose, and hs-CRP) pre-and post-intervention of the apple/berry juice were expressed as medians and interquartile ranges, as presented in Table 3. The changes observed for the anthropometric parameters were not statistically significant (*p* > 0.05), except for SMM (*p* = 0.025).

Although the decrease in the T-C level was slight, it was statistically significant (from 6.29 ± 0.99 to 5.94 ± 0.72 mmol/L). Similarly, a beneficial increase in HDL-C levels (from 1.30 ± 0.29 to 1.55 ± 0.32 mmol/L), Mg (from 0.85 ± 0.03 to 0.90 ± 0.05 mmol/L), and TAS (from 1.68 ± 0.08 to 1.81 ± 0.10 mmol/L), and a decrease in glucose (from 5.50 ± 0.72 to 5.24 ± 0.74 mmol/L) were observed post-intervention. 

The percentage change for the lipid profile (T-C, LDL-C, HDL-C, and TG) in the number of participants (*n* = 20) in plasma pre-and post-intervention of apple/berry juice, according to NCEP ATP III, is presented in Table 4. In general, participants’ lipid profiles improved as the proportion of women with optimum, near or above optimal, and borderline high values increased, while the proportion of participants with high and very high values declined—this is seen as positive in terms of reducing CVD risk factors.

In addition, there was a statistically significant improvement in the LDL/HDL ratio (from 3.40 ± 0.99 to 2.66 ± 0.63 mmol/L) in all women. Interestingly, the 13 women with the risk level of LDL/HDL ratio values in the pre-intervention (mean value 4.04 ± 0.61 mmol/L) showed significant improvements after the intervention (3.00 ± 0.47 mmol/L); two women reached the target value (<2.5 mmol/L), five women showed a level below the risk (<3.0 mmol/L), and six women were in the range of 3–4 mmol/L, while in the pre-intervention, these women showed values above 4 mmol/L. The changes in hs-CRP (inflammatory biomarker) in our monitored set of participants were not significant. 

### 3.4. Changes in Lipid Profile, hs-CRP, and TAS in Women with LDL Subclass Phenotype B

The average values of lipid profiles, hs-CRP, TAS, and the distribution of cholesterol in different lipoprotein subfractions in the women with LDL subclass phenotype B are given in Table 5. Atherogenic phenotype B is characterized by the presence of atherogenic LDL3-7 subfractions and was determined in six women before the intervention. However, after the intervention, all these women had non-atherogenic phenotype A. Indeed, there was a significant reduction in the level of atherogenic subfractions LDL3-7 from the initial value of 0.45 ± 0.19 to the post-intervention value of 0.09 ± 0.07 mmol/L. Although, the level of T-C and LDL-C remained unchanged, a significant increase in HDL-C was found after the intervention in all women, with an average value of 1.54 ± 0.26 (four participants reached the optimal reference value of HDL-C, ≥1.55 mmol/L). The hs-CRP did not change significantly. However, the TAS significantly increased in all women, with mean values of 1.72 ± 0.10 and 1.88 ± 0.13 mmol/L, at pre-and post-intervention, respectively. 

**Figure 2:**
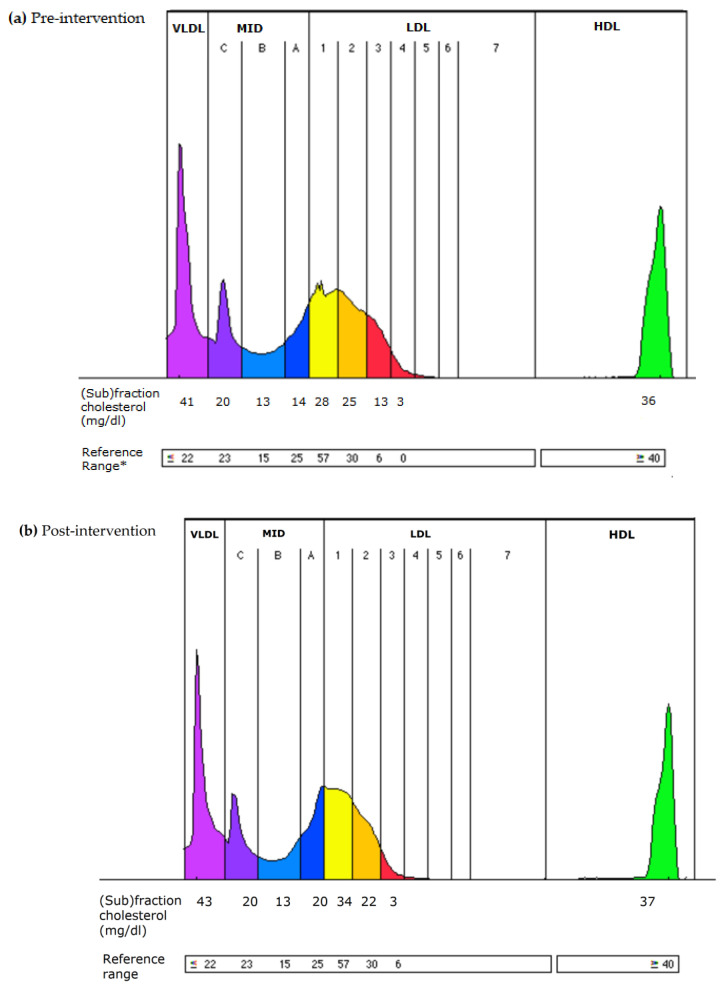
Changes in LDL lipoprotein subfractions in women with atherogenic phenotype B after the consumption of apple/berry juice for 6 weeks. (**a**) Pre-intervention: LDL phenotype B with atherogenic LDL3-7 subfractions (in red) and large-less atherogenic subfractions LDL1-2 in yellow; (**b**) post-intervention: LDL phenotype A almost without atherogenic LDL subfractions; LDL3-7 (in red) and large-less atherogenic subfractions LDL1-2 in yellow.

Figure 2 shows a representative lipoprotein profile of a woman with phenotype B in response to the intervention. At pre-intervention, the women had an optimal value of T-C (4.96 mmol/L) and LDL-C (2.97 and 2.92 mmol/L). However, the HDL-C (0.86 mmol/L) and the value of the LDL/HDL ratio (4.02 mmol/L) were out of the reference/optimal value, presenting an increased risk for CVD. The intervention with the apple/berry juice for 6 weeks significantly improved the level of atherogenic LDL3-7 subfractions (0.08 mmol/L), HDL-C (1.13 mmol/L), and LDL/HDL (3.07 mmol/L). 

## 4. Discussion

In the present study, the dietary intervention, involving 6 weeks’ consumption of 300 mL of a pure apple/berry juice, led to an increase in blood TAS and an improvement in lipid profiles through a significant reduction in the level of atherogenic subfractions LDL3-7, the LDL/HDL ratio, and an increase in HDL-C. By analyzing Table 4, the reader may encounter some beneficial modulations of T-C, LDL, and TG. Based on the overall percentual changes between the pre-and post-intervention, the proportion of women with optimum and near or above optimal values of T-C, LDL-C, and TG increased, whereas the number of women with high and very high values decreased.

The juice used in this intervention study was commercial high-quality juice (not from concentrates; no added sugar; without artificial sweeteners, colors, and preservatives); made of whole apples, chokeberries, cranberries, and blueberries. Several previous studies have provided evidence that the beneficial effect of fruit juices on cardiovascular health is associated with the presence of polyphenolic compounds in the fruit [42]. Bondonno et al. (2018) [43] demonstrated that the cardioprotective properties of apples are mainly due to flavonoids found in abundance in the apple skin [43]. Chokeberry, blueberry, and cranberry juice contain phenolic compounds with cardioprotective effects [44], including rutin, quercetin, chlorogenic acid, and others [45], which were also found in the apple/berry juice (Table 2). Indeed, the black chokeberry fruit has one of the greatest in vitro antioxidant activities among berry fruits and is one of the richest sources of edible phenolic compounds [46]. Based on the literature, the content of phenolics in chokeberries is reported to be more than 2000 mg/100 g [15], which is a much higher amount than of blueberries (258–531 mg/100 g), cranberries (120–709 mg/100 g), and apple (185–347 mg/100 g) [47,48]. The bioactive compounds in berries consist mostly of phenolic compounds (i.e., phenolic acids and flavonoids, such as anthocyanins, flavonols, and tannins) [29]; however, berries contain plenty of biologically active substances, such as a high concentration of vitamin C and a good source of mineral content (phosphorus, calcium, iron, potassium, magnesium, etc.) [48]. The combination of different berries provided a good source of various phenolics. In addition, further studies are needed to fully evaluate the mechanistic effects of such phenolic compounds on the associated antioxidant and anti-inflammatory effects in vivo, especially in terms of the concentrations to be set up for clinical trials [20,48]. Likewise, it is noteworthy to highlight that, according to some studies, it is preferable to consume fresh fruit or juice, instead of dehydrated forms, due to the oxidation of some phenolic compounds before uptake, with the consequence of a loss of bioactivity [43].

Furthermore, the apple/berry juice exerted a beneficial effect on the modulation of cholesterol in overweight/obese women, as evidenced by our results in Table 3 and Table 4. The blood serum levels of T-C decreased (*p* = 0.015) and HDL-C significantly increased (*p* < 0.001) in the monitored group after the 6-week consumption of apple/berries juice. The proportion of women with optimum, near-optimal, or borderline high lipid profiles (T-C, LDL-C, HDL, TG) generally increased, whereas the proportion of participants with high or very high values decreased (Table 4), which is considered positive in terms of reducing CVD risk. HDL-C and LDL-C play opposing roles in controlling body cholesterol; both reduced depositions (LDL reduction) and increases in removals (raised HDL) can improve CVD [49]. Huang et al. (2016) [50], in their review, point to a significant reduction in LDL cholesterol, as well as systolic blood pressure, fasting glucose, and BMI under the influence of berry consumption [50]. Blueberry and blackcurrant extract significantly reduced T-C and LDL-C levels after four weeks of consumption [51]. Another study conducted by Koutsos et al. (2020) [52] also revealed that the consumption of two apples a day allowed for reduced serum cholesterol levels and improved some cardiometabolic biomarkers in hypercholesterolemic adults [52]. The LDL-C and HDL-C levels are routinely measured in clinical practice for screening for CVD risk in individuals [53]. However, the LDL/HDL cholesterol ratio has been suggested as a more reliable indicator of cardiovascular risk than the isolated parameters, particularly LDL [40]. Kunutsor et al. (2017) [53] found that the LDL/HDL ratio had links to an increased risk of sudden cardiac death [53]. It has been recommended that individuals with a high LDL/HDL ratio (>3 mmol/L) should begin treatment due to abnormal cholesterol levels [40]. The apple/berry juice intervention significantly improved (*p* < 0.001) the LDL/HDL ratio in all women (from 3.56 mmol/L to 2.67 mmol/L), being close to the target value of 2.5 mmol/L. 

Recently, studies support that sdLDL is a good CVD risk biomarker [54,55,56] and is strongly associated with atherosclerotic risk markers, such as inflammation, thrombosis, hematological markers, and prediabetes [55,56]. To identify and quantify atherogenic (LDL3-7) and non-atherogenic sdLDL subfractions (LDL1 and LDL2) in plasma, a polyacrylamide gel electrophoresis (Lipoprint system) was used. The presence of atherogenic LDL3-7 was detected in six women at baseline. The important finding was a significant decrease in the level of atherogenic LDL3-7 from the initial value of 0.45 ± 0.19 to 0.09 ± 0.07 mmol/L (*p* < 0.003) in all women in post-intervention, independent of LDL cholesterol (*p* > 0.05) or TG (*p* > 0.05). Moreover, the HDL-C significantly increased and consequently improved the LDL/HDL ratio from the risk value (3.61 ± 1.14) to close to the target value (<2.5 mmol/L), being 2.61 ± 0.52 mmol/L after the intervention (Table 5), which significantly contributed to the improvement in their cardiovascular health. The presence of sdLDL influences many aspects of atherosclerosis, such as affecting lipid metabolism, promoting the release of inflammatory factors leading to inflammatory reaction, and releasing excessive ROS and RNS to produce oxidative stress [57]. Inflammatory biomarker Hs-CRP can be used to measure the association between sdLDL and low-grade inflammation [55] and to predict long-term cardiovascular risk [58]. The apple/berry juice intervention did not significantly alter hs-CRP and the women in the monitoring group presented a low-average risk (<3 mg/L). The selection of optimal therapeutic measures, including the removal of atherogenic lipoproteins, is crucial as part of a comprehensive therapeutic approach. The identification of the type of lipoprotein profile (atherogenic vs. non-atherogenic) represents a benefit in lipid diagnostics, advances the quality of interpretation in lipoprotein analysis [59], and may help identify individuals at a higher risk of developing CVD [14]. It turns out that the consumption of fruits rich in polyphenols in the form of 100% juice can be a suitable therapeutic strategy in the management of the primary prevention of lipid profile disorders.

In addition, the effect of apple/berry juice has been tested on anthropometric parameters (Table 3). The 6-week consumption of apple/berry juice improved the SMM (*p* = 0.025). Recent research shows that the antioxidant and anti-inflammatory properties of phenolic compounds can contribute to the reduction in muscle atrophy induced by oxidative stress and inflammation [60]. The antioxidant and radical-scavenging properties of black chokeberry extract have been attributed to its high antioxidant content (total phenolics, total flavonoids, and proanthocyanidin content), which protects the body against damage from reactive oxygen radicals [61]. Pedersen et al. (2000) [62] reported that cranberry juice (500 mL) increased the ability of plasma of healthy female volunteers to reduce oxidative stress [62]. We found a significant increase in plasma antioxidant status (TAS, *p* = 0.001) and magnesium level (*p* = 0.001) after the intervention in all participants. Given these results, we hypothesize that apple/berry juice may be beneficial for preventing skeletal muscle aging due to its rich concentration of biologically active substances, such as antioxidant phenolics and other minor compounds. Although the women in this study had fasting blood glucose levels in the normal range (4.0–5.5 mmol/L), the intake of apple/berry juice slightly but significantly decreased glucose values, thus, making the consumption of apple/berry juice beneficial for weight maintenance. 

This study has certain limitations. The major limitation is the lack of the control group treated with the placebo and/or lack of the control experiment in the same studied group before the treatment with the juice. The thermal pasteurization of the juice may decrease the content of bioactive compounds, such as vitamin C and some phenolics, thus, other methods of pasteurization and/or the control of juice without treatment are of further interest. In addition, flavonoid intake from the background diet was not assessed. Another two limitations may be the small number of participants, which could reduce the statistical power to detect differences in the results, and the short duration of this intervention study. 

## 5. Conclusions

The consumption of apple/berry juice with a high content of bioactive substances such as phenolic compounds led to an increase in TAS and the positive modulation of the TC, HDL-C, LDL/HDL ratio in overweight/obese women at risk of cardiovascular diseases. We evaluate, very positively, the decrease in atherogenic sdLDL (LDL3-7 subfractions) in all women. The presence of sdLDL in blood plasma appears to be a significant predictor of the atherosclerosis process and CVD risk. The present study shows the possible prevention of CVD and positive modulation of the lipid profile of women at risk of cardiovascular diseases in a non-pharmacological way. 

These findings are interesting both from a clinical and commercial point of view, as they could not only lead to alternative, more natural therapies for CVD prevention, but also to the development of new functional products. Future studies should focus on the optimization of beneficial doses of juices that are rich in bioactive substances, with a larger number of participants, control placebo group, and a longer duration of the study. Additionally, further research is needed, aiming at a detailed study of the mechanisms of the atherogenicity of lipoprotein subfractions, as well as research on the gentle methods of the pasteurization of juices.

## Figures and Tables

**Table 1:** Characteristics of the selected participants at baseline.

Parameters	Standard/Optimal Range	Women (*n* = 20) Baseline		
		Mean ± SD	Max	Min
Age (years)		52.95 ± 5.80	60	41
Bodyweight (kg)		75.25 ± 8.34	100.40	57.20
BMI (kg/m^2^)	18.5–24.9	28.01 ± 2.88	37.33	25.05
PBF (%)	18–28	38.35 ± 5.05	48.81	28.60
WHR	0.75–0.85	0.97 ± 0.05	1.08	0.90
SMM (kg)	31.7–38.7	25.52± 2.81	30.06	20.84
Systolic blood pressure (mm Hg)	˂120	137.40 ± 16.75	169	115
Diastolic blood pressure (mm Hg)	˂80	81.95 ± 9.90	99	60
T-C (mmol/L)	˂5.19	6.29 ± 0.99	8.51	4.94
TG (mmol/L)	˂1.7	1.47 ± 0.66	2.42	0.44
HDL-C (mmol/L)	≥1.55	1.30 ± 0.29	1.83	0.86
LDL-C (mmol/L)	˂2.6	4.19 ± 0.83	6.03	3.12
LDL/HDL (mmol/L)	˂2.5	3.40 ± 0.99	5.15	2.04
hs-CRP (mg/L)	˂1	1.90 ± 2.35	10.78	0.36
Glucose (mmol/L)	4.0–5.5	5.50 ± 0.72	7.35	4.51

Abbreviations: BMI, body mass index; PBF, percent of body fat; WHR, waist-hip ratio; SMM, skeletal muscle mass. Values of blood pressure (systolic and diastolic) were compared with reference values of American College of Cardiology/American Heart Association guidelines [39]; values of T-C, HDL-C, LDL-C, and TG were compared with reference values of the NCEP ATP III [12]. The optimal/target value of the LDL/HDL ratio < 2.5 mmol/L is the value for women at primary prevention (women with LDL/HDL above 3.0 mmol/L) [40]. Hs-CRP values <1 mg/L, 1–3 mg/L, and >3 mg/L indicate low, average, and high relative cardiovascular risk, respectively [41]. Baseline refers to the number of participants (*n* = 20) before intervention. Data are presented as mean ± SD.

**Table 2:** The concentration of the antioxidant phenolic compounds in the apple/berry (chokeberries, blueberries, cranberries) juice used in the present study.

Parameter	Units	Results
TPC	µg/mL	286.25 ± 8.41
TA	µg/mL	7.82 ± 0.93
Rutin	µg/mL	64.64 ±7.12
Quercetin	µg/mL	20.77 ± 0.80
Kaempferol	µg/mL	0.41 ± 0.03
Chlorogenic acid	µg/mL	27.47 ± 2.29
Gallic acid	µg/mL	6.80 ± 0.62
Rosmarinic acid	µg/mL	1.24 ± 0.11
Caffeic acid	µg/mL	1.31 ± 0.12
Antioxidant activity (AA), inhibition of DPPH	%	73.24 ± 1.85

The total phenolic content (TPC) is expressed as the mg of gallic acid equivalent (GAE), while total anthocyanins (TA) content is expressed as the mg of cyaniding-3-glucoside equivalent (CGE). Data are presented as mean ± standard deviation (SD) (for each sample, *n*= 4).

**Table 3:** Changes in anthropometric parameters, blood pressure, and plasma parameters of overweight/obese women with associated CVD risk factors after (post) the apple/berry juice intervention.

Parameters	Overweight/Obese Women with Associated CVD Risk Factors (*n* = 20)		
	Pre	Post	*p*-Value
*Anthropometric parameters*			
Body weight (kg)	74.30 (69.35–79.65)	75.55 (69.68–81.23)	NS
BMI (kg/m^2^)	27.79 (25.86–28.91)	27.61 (25.87–28.97)	NS
WHR	0.96 (0.92–0.99)	0.97 (0.92–0.99)	NS
PBF (%)	38.16 (34.80–42.47)	37.47 (33.59–41.01)	NS
SMM (kg)	25.74 (23.22–27.65)	25.89(23.24–27.93)	0.025
*Blood pressure*			
Systolic blood pressure (mm Hg)	135 (124–150)	131 (116–142)	NS
Diastolic blood pressure (mm Hg)	82 (75–90)	79 (74–85)	NS
*Blood lipids*			
T-C (mmol/L)	6.15 (5.59–6.82)	6.01 (5.39–6.34)	0.015
LDL-C (mmol/L)	4.00 (3.55–4.77)	3.83 (3.48–4.26)	NS
HDL-C (mmol/L)	1.27 (1.12–1.59)	1.51 (1.35–1.75)	<0.001
LDL/HDL (mmol/L)	3.56 (2.41–4.26)	2.67 (2.28–3.11)	<0.001
TG (mmol/L)	1.60 (0.87–2.05)	1.46 (0.95–1.81)	NS
*Other biochemical parameters*			
Mg (mmol/L)	0.85 (0.82–0.87)	0.91 (0.88–0.93)	0.001
TAS (mmol/L)	1.68 (1.62–1.73)	1.79 (1.75–1.85)	<0.001
Glucose (mmol/L)	5.29 (5.08–5.82)	5.21(4.84–5.59)	<0.041
hs-CRP (mg/L)	1.13 (0.62–2.11)	1.14 (0.70–1.85)	NS

*n*, number of participants; pre, parameters analyzed before the study; post, parameters analyzed at the end of the study. Data are expressed as medians and interquartile ranges in parentheses. Statistical significance at *p* < 0.05.

**Table 4:** Distribution percentage changes (%) of lipid profiles in participants pre- and post-intervention of apple/berry juice according to reference values of NCEP ATP III.

Reference Values of NCEP ATP III		Participants (%)	
		Overweight/Obese Women with CVD Risk Factors (*n* = 20)	
		Pre	Post
T-C			
Desirable	<5.19 mmol/L	10	20
Higher borderline	5.2–6.19 mmol/L	45	50
High	≥6.2 mmol/L	45	30
LDL-C			
Optimal	<2.6 mmol/L	0	0
Near or above optimal	2.6–3.3 mmol/L	10	10
Higher borderline	3.4–4.1 mmol/L	50	60
High	4.2–4.9 mmol/L	15	25
Very high	>4.9 mmol/L	25	5
HDL-C			
High	≥1.55 mmol/L	30	50
Higher borderline	1.04–1.54 mmol/L	50	45
Low	<1.03 mmol/L	20	5
TG			
Optimal	<1.7 mmol/L	60	65
Higher borderline	1.70–2.25 mmol/L	30	25
High	2.26–5.64 mmol/L	10	10
Very high	≥5.65 mmol/L	0	0

Values of T-C, HDL-C, LDL-C, and TG were compared with reference values of the NCEP ATP III [12].

**Table 5:** Lipid profile, lipoprotein subfractions, and inflammatory biomarkers (hs-CRP) before (pre) and after (post) 6 weeks of the apple/berry intervention in overweight/obese women with atherogenic phenotype B (presence of LDL3-7 subfractions).

Parameters	Overweight/Obese Women with Phenotype B (*n* = 6)		
	Pre	Post	*p*-Value
T-C (mmol/L)	6.41 ± 1.35	5.89 ± 0.56	NS
LDL-C (mmol/L)	4.32 ± 1.10	3.94 ± 0.43	NS
HDL-C (mmol/L)	1.24 ± 0.26	1.54 ± 0.26	0.002
LDL/HDL (mmol/L)	3.61 ± 1.14	2.61 ± 0.52	0.032
TG (mmol/L)	1.51 ± 0.83	1.31 ± 0.57	NS
VLDL (mmol/L)	1.45 ± 0.49	1.18 ± 0.15	NS
IDL1 (mmol/L)	0.74 ± 0.22	0.55 ± 0.08	NS
IDL2 (mmol/L)	0.60 ± 0.21	0.59 ± 0.21	NS
IDL3 (mmol/L)	0.59 ± 0.23	0.75 ± 0.18	0.029
LDL1 (mmol/L)	0.95 ± 0.23	1.06 ± 0.27	NS
LDL2 (mmol/L)	1.00 ± 0.32	0.72 ± 0.18	0.012
LDL3-7 (mmol/L)	0.45 ± 0.19	0.09 ± 0.07	0.003
hs-CRP (mg/L)	1.21 ± 1.23	1.26 ± 1.01	NS
TAS (mmol/L)	1.72 ± 0.10	1.88 ± 0.13	0.005

*n*, number of women with atherogenic subfractions LDL3-7 in blood plasma at the beginning of the study; data are presented as mean ± SD. Statistical significance at *p* < 0.05.

## Data Availability

All datasets related to the results of this study are available from the primary author on request.
